# Oxytocin and Bone: Review and Perspectives

**DOI:** 10.3390/ijms22168551

**Published:** 2021-08-09

**Authors:** Véronique Breuil, Marie-Charlotte Trojani, Amri Ez-Zoubir

**Affiliations:** 1 Faculté de Médecine Nice, UMR E-4320 TIRO-MATOs CEA/DRF/Institut Joliot, Université Côte d’Azur, CEDEX 2, 06107 Nice, France; mctroj@hotmail.fr; 2 French National Centre for Scientific Research (CNRS), Inserm, iBV, Université Côte d’Azur, CEDEX 2, 06107 Nice, France; Ez-Zoubir.Amri@unice.fr

**Keywords:** oxytocin, bone cells, osteoporosis, pharmacological perspectives

## Abstract

Recent data demonstrate the anabolic effect of oxytocin on bone. Bone cells express oxytocin receptors. Oxytocin promotes osteoblasts differentiation and function, leading to an increased bone formation with no effect on bone resorption and an improvement of bone microarchitecture. Oxytocin is synthetized by osteoblasts, and this synthesis is stimulated by estrogen. Animal studies demonstrate a direct action of oxytocin on bone, as the systemic administration of oxytocin prevents and reverses the bone loss induced by estrogen deficiency. Although oxytocin is involved in bone formation in both sexes during development, oxytocin treatment has no effect on male osteoporosis, underlining the importance of estrogen that amplifies its local autocrine and paracrine secretion. There are few human data showing a decrease in the oxytocin serum level in anorexia nervosa independently of estrogen and in amenorrheic women associated with impaired bone microarchitecture; in post-menopausal women a higher oxytocin serum level is associated with higher bone density, but not in osteoporotic men. Oxytocin displays many effects that may be beneficial in the management of osteoporosis, cardiovascular diseases, cognitive disorders, breast cancer, diabetes and body fat gain, all age-related diseases affecting elderly women, opening exciting therapeutic perspectives, although the issue is to find a single route, dosage and schedule able to reach all these targets.

## 1. Introduction

Oxytocin (OT), also called “the love hormone”, has long been recognized for its role in parturition, milk ejection, reproduction and social behavior, but it is only recently that growing evidence for its role in bone metabolism has arisen [1]. Indeed, OT appears to be involved in the regulation of the following two out of the three components of body composition: bone and fat [2]. OT is mainly synthesized by hypothalamic neurons but is also synthesized in peripheral tissues, e.g., uterus, placenta, amnion, corpus luteum, testis, heart and osteoblasts [3,4,5].

Osteoporosis is the most common metabolic skeletal disorder, characterized by a decrease in bone mass and alterations of the microarchitecture, leading to skeletal fragility and an increased risk of fractures [6]. Bone mass is maintained by the balance between bone formation assumed by osteoblasts, which share a common mesenchymal precursor with adipocytes, and bone resorption assumed by osteoclasts [7]. With aging, the composition of bone marrow shifts to an increase in adipocyte formation, decrease in osteoblast functions and a major increase in osteoclast activity, resulting in osteoporosis [7]. Osteoporosis is a multifactorial disease with risk factors including aging, gonadal insufficiency, glucocorticoids and diabetes [7]. In Western populations, the risk of osteoporotic fracture occurring in the remaining lifetime after 50 year old individuals is 50% for women and 20% for men and, thus, is recognized as a major public health problem [8].

In this review, we will present current data on the role of OT in bone metabolism and osteoporosis and the potential future applications of OT as a therapeutic target in the treatment of osteoporosis, post-menopausal or secondary causes of osteoporosis and, more generally, in menopause-related diseases.

## 2. Oxytocin and Bone Cells

The first evidence that OT may be involved in bone metabolism emerged in the late 90 s when it was shown that bone cells, osteoblasts and osteoclasts express OT receptors [9,10].

### 2.1. Oxytocin and Osteoblasts

OT negatively modulates adipogenesis while promoting osteoblast differentiation and function, leading to an increased bone formation [11,12]. Indeed, Elabd C et al. showed that OT receptor mRNA levels decrease during adipogenesis and increase during osteogenesis, and that OT treatment of human bone marrow-derived mesenchymal stromal cells enhanced osteoblast differentiation and reduced adipocyte differentiation [11]. According to these data, Tamma R. et al., using a mouse model of OT deletion (OT^−/−^), showed in an ex vivo culture of calvaria that mineralization was dramatically reduced in osteoblasts deleted for the OT gene and partially rescued by an exposure to recombinant human OT [12]. At the molecular level, OT increases osteoblasts’ development toward a mineralizing phenotype by inducing an upregulation of BMP-2 through the schnurri-2 and ATF-4 pathways [12,13]. The anabolic action of OT appears to be related in part to a direct effect on its receptor expressed on osteoblasts, inducing its nuclear localization in osteoblasts [5,12].

**Figure 1:**
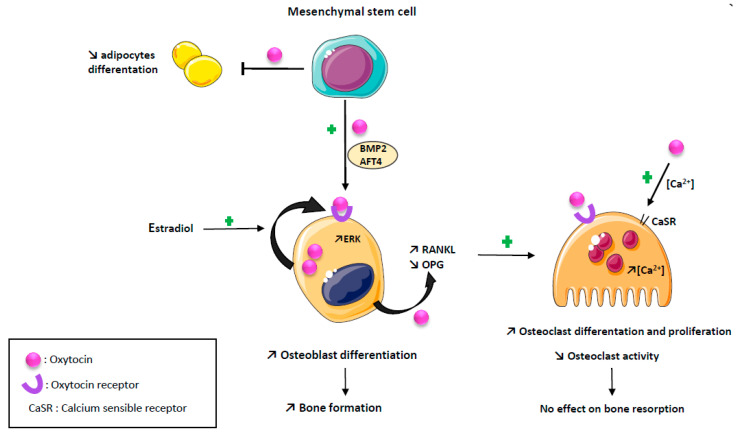
Effects of oxytocin on bone cells. Oxytocin has an anabolic action promoting osteoblast differentiation and function by an upregulation of BMP2 and AFT4 pathways, and reducing adipogenesis. Estradiol positively stimulate oxytocin production by osteoblast through an activation of the MAP kinase/ERK pathway and oxytocin receptor expression, synergistic with a local feed-forward loop. Oxytocin has a paradoxical effect on osteoclast resulting in no effect on bone resorption: osteoclast number is increased by promoting RANKL expression and decreasing OPG expression; mature osteoclast resorption is decreased by supporting intracellular calcium concentration.

Estrogens are known to stimulate OT synthesis in bone as in other tissues [13,14,15]. Estrogen positively stimulates OT production by osteoblasts, through an activation of the MAP kinase/ERK pathway and OT receptor expression by a genomic mechanism of action [16]. These two effects are synergistic through a local feed–forward loop, as there is an autocrine/paracrine secretion of OT by osteoblasts induced by estrogen [16,17,18]. The effects of OT on osteoblasts are summarized in Figure 1.

### 2.2. Oxytocin and Osteoclasts

OT both reduces osteoprotegerin expression and increases RANKL expression by osteoblast, thus, promoting osteoclast differentiation [12,19]. However, although in an in vitro culture of osteoclasts, the precursors treated by OT the number of osteoclasts were increased, their ability to resorb bone was diminished [12]. The decrease in the bone resorption capacity of osteoclasts induced by OT is explained by the ability of OT to increase intra-cellular calcium in osteoclasts known to diminish osteoclast function by increasing NO production [12]. Indeed, OT-induced signaling cascades stimulate the calcium release from intracellular stores and triggered ERK signaling both in osteoblast and osteoclast, but the intra-cellular calcium increase is a single spike in osteoblast, returning immediately to baseline, while it is a slower but long lasting calcium increase in osteoclasts [12,13]. Reinforcing these data, the OT treatment of ovariectomized mice partially restores the decrease in the osteoclasts number, increases TNFα mRNA expression and stimulates osteoclastogenesis through an increase in the RANKL/osteoprotegerin ratio produced by mesenchymal stem cells [20]. Altogether, these data demonstrate that, although OT stimulates osteoclasts differentiation, it also inhibits the activity of mature osteoclasts, resulting in no effect on bone resorption [3,12,19]. This paradoxical effect is explained by a down regulation of OT receptors on bone cells [12,19]. In summary, the action of OT on bone metabolism is mainly an anabolic action mediated by osteoblast. The effects of OT on osteoclasts are summarized in Figure 1.

## 3. Oxytocin and Bone: Animal Studies

### 3.1. OT and Bone Cells

Using two different approaches, animal studies demonstrate that OT is involved in the pathophysiology of bone remodeling. In agreement with the in vitro data on the role of estrogen in the regulation of OT secretion, ovariectomy, used as an animal model mimicking post-menopausal osteoporosis, induces a significant decrease in OT plasma levels in mice (−40%) and rats (−35%) [11]. OT and OT-receptor knock-out mice develop osteoporosis characterized by a defect in bone formation related to a decrease in osteoblastic differentiation that could be rescued by an intra-peritoneal injection of OT [12]. In ovariectomized rats, a daily intra-peritoneal injection of OT prevents the decrease in osteoblasts and osteocytes numbers, of the osteoprotegerin/RANKL serum ratio and the increase in bone turnover markers [20]. Contrarily, an intracerebroventricular OT infusion did not affect serum bone turnover markers nor ex vivo osteoblast or osteoclast formation, underlying the fact that OT bone effects are related to its peripheral action but not by an indirect effect on the neuropituitary hormones known to regulate bone remodeling [12]. Taken together, all these data show that OT has a direct action on the skeleton that appears to be related predominantly to a peripheral action of OT more than an indirect action through the central nervous system.

### 3.2. OT and Bone Microarchitecture

At the tissue level, the OT treatment improved bone microarchitecture. Indeed, subcutaneous OT injection prevents and reverses bone loss in ovariectomized mice by enhancing the bone microarchitecture assessed using a micro-CT (increase in total bone volume, trabecular number and decrease in trabecular spacing) and biomechanical strength (stiffness, ultimate force, Young’s modulus and ultimate stress, although cortical stiffness remains lower than in sham mice) [11]. In ovariectomized rats, a daily intra-peritoneal injection of OT induces a beneficial effect on the trabecular bone parameters mediated through the restoration of osteoblast/osteoclast cross talk via the RANKL/osteoprotegerin axis [20]. These beneficial effects on the bone density and the microarchitecture of OT systemic administration have been confirmed by other studies in ovariectomized rats and rabbits [21,22].

The action of OT on bone metabolism differs according to gender. The deletion of OT or the OT receptor causes osteoporosis both in male and female mice by decreasing bone formation [12]. However, although OT treatment reverses osteoporosis in ovariectomized mice, this effect is not observed in orchidectomized mice, demonstrating that OT reverse features of hypogonadism are directly related to estrogen pathways, which amplify the local autocrine/paracrine secretion of OT by osteoblasts [23]. These data suggest that OT acts on bone by a direct effect on OT receptors on bone cells and is able to reverse the features of hypogonadism directly related to estrogen pathways but is not able to reverse the osteoporosis related to androgen deficiency [11,12,23].

One of the challenges of fractures treatment is to assure bone healing as soon as possible. In this context, the anabolic properties of OT have been tested to determine whether it could accelerate bone healing locally. The systemic administration of OT promotes peri-implant bone healing and osseointegration of titanium implant in ovariectomized rats, increasing bone volume and improving trabecular micro-structure surrounding the implant [24]. Furthermore, Aykay AS et al. showed, in a rat model, that a thermosensitive hydrogel graft incorporating OT-loaded PGLA microspheres provides an accelerated bone regeneration in the rat calvaria [25].

### 3.3. OT and Bone Marrow Adiposity 

It is recognized that there is an inverse relationship between bone marrow adiposity and bone mass. Indeed, it has been shown that osteoporosis is associated with a gain in bone marrow adiposity, related to an increased formation of adipocytes from mesenchymal stem cells at the expense of osteoblasts [26,27,28]. Marrow fat content increases with trabecular microarchitecture deterioration and is connected to the prevalence of bone fracture in osteoporosis [26,27,28]. In ovariectomized mice, a subcutaneous injection of OT reverses bone loss assessed using micro-computed tomography and reduces bone marrow adiposity by decreasing marrow adipocyte density [11]. In an ovariectomized rabbit, an early in vivo subcutaneous injection of OT slowed down bone deterioration and reduced bone marrow adiposity accumulation assessed using 3.0-T magnetic resonance spectroscopy and micro-computed tomography [29].

## 4. Oxytocin and Osteoporosis: Human Data

There are only a few human data available on the relationship between OT and bone summarized in Table 1.

According to animal studies, in a cross-sectional analysis, Maestrini S. et al. showed that plasma OT levels are significantly decreased after menopause, and this decrease is more important in obese women than in normal weight women [35]. In postmenopausal women, without previous or current diseases associated with secondary osteoporosis and/or previously or current treatment for osteoporosis, divided into two groups, a control group (no fractures and normal bone mineral density (BMD)) and a group with severe osteoporosis (at least one osteoporotic fracture and low BMD values)), circulating OT levels were significantly lower in osteoporotic women, in agreement with the rodent data [11]. In this population, OT serum levels were not correlated to any other measured neuropituitary hormones, including leptin and estradiol, and logistic regression analysis showed that osteoporosis status remained significantly correlated to OT serum levels, regardless of age [31]. These data reinforce the fact that the anabolic effect of OT on bone is predominantly related to a direct and peripheral action on bone cells. In the OPUS, including 1097 post-menopausal women, cohort, higher OT serum levels were associated with lower bone turnover markers and a higher BMD, especially at the hip in women with low estradiol or high leptin serum levels, reinforcing the fact that OT has a direct effect on bone cells independently of estradiol OT mediated action [33]. However, in this study, there was no significant relationship between OT serum levels and osteoporotic fractures: 313 fractures were observed in the population, and half of them were observed in subjects with BMD above the diagnostic threshold of osteoporosis precluding any definite conclusion [33]. In line with animal studies regarding the sex specific action of OT, in a large prospective cohort of men (MINOS), OT serum levels were not associated with BMD, bone turnover rate or prevalent fractures [34]. This could reflect the major role of estrogens deficiency in the pathophysiology of osteoporosis in women compared to men, although estrogens also participate to the regulation of bone metabolism in men [37,38]. Nevertheless, in the MINOS cohort, there was a weak significant trend for a higher fracture risk in men with a low serum OT level after adjustment for confounders including BMD and serum OT was significantly lower in men with severe osteoporosis compared to men with a normal bone status, suggesting effects of OT on other determinants of fracture risk such as muscle strength [34]. However, in another cross-sectional study including 37 hypopituitary men without any previous history of fracture or any medication for osteoporosis, there was a robust positive association between fasting OT serum levels and BMD (Z-scores at the lumbar spine, femoral neck and total hip) and favorable hip geometry and strength variables at the intertrochanteric region in men with central diabetes insipidus but not in men with anterior pituitary deficiencies [36]. 

Anorexia nervosa is a classic cause of secondary osteoporosis related to low body weight and decreased gonadal function [39]. In a study of 36 women suffering from anorexia nervosa compared to 19 healthy controls, OT serum levels were 50% lower in anorexic women, remaining significant after controlling for estradiol levels, and significantly associated with a low BMD [30]. According to these data, amenorrheic athletes have a decrease in nocturnal OT secretion that correlated strongly with impaired cortical and trabecular microarchitecture, independently of estradiol serum levels, and accounted for a substantial portion of the variability in microarchitectural and strength parameters in this population [32].

## 5. Oxytocin as a Therapeutic Agent: Perspectives

As OT has pleiotropic effects, the therapeutic perspectives are truly exciting. Indeed, OT has broad implications for general health: OT is a stress-coping molecule with anti-inflammatory and antioxidant properties; influences the immune system, body composition, cognitive functions and mood; and has been tested in the treatment of numerous diseases, including anxiety, pain, diabetes, cardiovascular diseases and breast cancer [1,27,40,41,42,43,44,45].

Currently, apart from animal studies, OT has not been tested as a treatment of osteoporosis in humans. There are various treatments available in osteoporosis that have demonstrated their efficacy to reduce osteoporotic fracture. Among them, bisphosphonates, denosumab, teriparatide and romosozumab (an sclerostin antibody) have a specific action on bone with a significant decrease in all types of fractures, but no extra skeletal beneficial effect on the medical conditions associated with the causes of osteoporosis [6]. Raloxifene, a selective estrogen receptor modulator, preserves bone mass and decreases vertebral fractures but not non-vertebral fractures; although raloxifene displays extra-skeletal benefits such as favorable effects on the lipid profile and risk of invasive breast cancer, it might worsen hot flashes, carries an estrogen-like risk of venous thrombosis, and increases the risk of death from stroke in women with cardiovascular risk factors [6,46]. Estrogen therapy prevents bone loss in postmenopausal women and reduces the risk of vertebral and hip fractures and is efficient on menopausal symptoms [6,47]. Estrogen therapy side effects include cardiovascular events (if initiated more than 10 years beyond menopause), thromboembolic disease, stroke and breast cancer [47]. Therefore, hormone replacement therapy is used for bone protection and fracture prevention only in the context of a primary indication for menopausal symptom treatment in recently post-menopausal women at low risk of cardiovascular disease, thromboembolic disease and breast cancer [47].

Menopause is associated with the following numerous health concerns: increased risk of osteoporosis, metabolic changes that predispose to cardiovascular disease and diabetes, cognitive decline, disrupted sleep and depressive symptoms [48,49]. Moreover, a reduction in estrogen levels leads to an alteration of the energy homeostasis, regulation of hunger and satiety signals resulting in a higher body mass index with a redistribution of fat mass from the subcutaneous to visceral adipose tissue compartment [50,51,52,53]. Currently, estrogen therapy is not recommended for the primary or secondary prevention of coronary heart disease or dementia [48].

**Figure 2:**
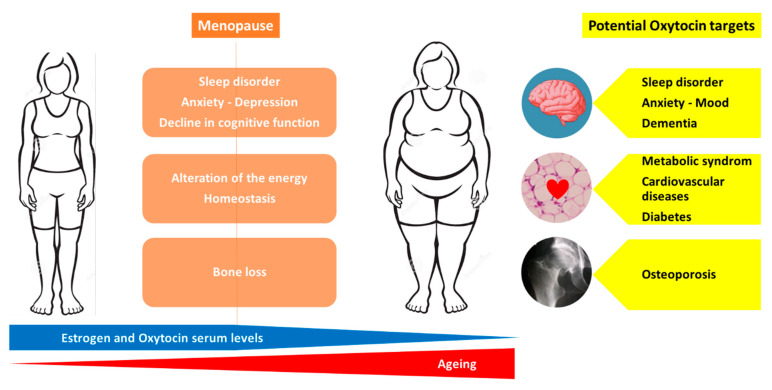
Potential therapeutic targets of oxytocin in menopause-related diseases.

Thus, obtaining a molecule that is able to target overall age-related diseases in women would be a dream. As previously discussed, an injection of OT (intra-peritoneal or sub-cutaneous) preserves and reverses the bone loss induced by estrogen deficiency in animal models [11,21,23,29,54]. Animal models of osteoporosis are not suitable to directly evaluate the efficacy on fracture risk of anti-osteoporotic molecules but use BMD, bone microarchitecture parameters and ex vivo bone strength tests as surrogate markers that are known to predict fracture risk. Thus, clinical studies are urgently required to evaluate the effect of OT on bone loss and fracture risk in post-menopausal women. OT is involved in cardiovascular regulation and protection, blood pressure regulation and exerts robust anti-oxidative and anti-inflammatory effects on cardiomyocytes [40]. Animal studies indicate that OT is not only a cardiovascular protective peptide but also critical for cardiovascular homeostasis and for the reduction in the severity of cardiovascular pathologies: OT treatment improves cardiac work, reduces apoptosis and inflammation and increases scar vascularization [40]. Menopause is associated with an increased risk of metabolic syndrome related to an increase in visceral obesity, a strong determinant of insulin resistance and recognized as a risk factor for cardiovascular diseases [55]. OT is involved in the regulation of eating behavior, energy expenditure, lipid metabolism and glucose homeostasis [56]. Indeed, OT preferentially suppresses the intake of sweet-tasting carbohydrates, both by actions on the brain’s reward systems and by effects on sweet taste receptors, while improving glucose tolerance [3]. A recent review by Lawson E et al. summarized the current data on the effects of OT on energy metabolism: OT increases lipolysis and reduces fat mass, including metabolically unfavorable visceral fat depots [56]. In animal studies, the peripheral administration (sub-cutaneous or intra-peritoneal) of OT reverses ovariectomy-induced body fat gain, increases markers of lipolysis and reduces fat mass, including metabolically unfavorable visceral fat depots [20,56,57]. In humans, the intra-nasal administration of OT increases fat utilization and reduces waist circumference [56]. Both central and peripheral OT treatments induce body weight loss in obese animal models with impaired leptin signaling, in diet-induced obesity rhesus monkeys and in obese humans [56]. Cognitive impairment, observed in stress disorders, and dementia are also a challenge in pathological ageing. OT is recognized to play a role in social behavior and stress coping, is involved in the regulation of hippocampal plasticity and memory formation, and increases the functional activity and connectivity in frontal regions associated with the impairment in autism [41,58]. It has been reported that the OT receptors’ polymorphisms are associated with impaired memory in humans, and OT knockout in mice displays memory deficit [43]. A systematic review of the effects of OT treatment in frontotemporal dementia reports an improvement in cognition and behavioral symptoms with a good tolerance [58]. Recent studies suggest neuroprotective effects of OT through the inhibition of the production of pro-inflammatory mediators [59]. Moreover, recent data point out the potential beneficial action of OT in vascular dementia, requiring further studies [42]. Finally, although some studies report that OT receptors may play a role in breast cancer development and progression and that several breast cancer cell lines express OT receptors, there is emerging evidence that OT may lower the risk of breast cancer and that OT receptors represent a promising therapeutic target for breast cancer [45]. Although these exciting findings warrant further investigations, at least OT did not increase the risk of breast cancer, in contrast to estrogen therapy. Thus, OT appears to be a molecule that could simultaneously target most of the health concerns related to menopause and ageing, namely osteoporosis, metabolic syndrome, body weight gain, cardiovascular diseases, stress, mood, dementia and breast cancer. Figure 2 represents the health issues related to menopause and the potential OT targets to prevent or treat these conditions.

Regarding secondary types of osteoporosis, glucocorticoid treatment and diabetes are the most common causes. Glucocorticoid and diabetes induced osteoporosis share pathophysiological characteristics: a predominant decrease in bone formation rather than an increase in bone resorption and an increase in bone marrow adiposity [60,61,62]. In glucocorticoid-induced osteoporosis, after an early transient increase in bone resorption, there is a long-term suppression of bone formation related to an increased sclerostin secretion and up-regulation of peroxisome proliferator-activated receptor that favor the differentiation of pluripotent precursor cells to adipocytes rather than osteoblasts, contributing to bone marrow adipocyte expansion [63,64]. Fragility fractures are now recognized as an important complication of both diabetes mellitus type 1 and type 2, particularly in those with long-term disease, poor glycaemic control, β cells failure and insulin treatment [65]. The mechanisms involved in type 1 diabetes imply a reduced BMD as a consequence of insufficient anabolic tone from insulin, while in type 2 diabetes, BMD is normal or high, but there is an alteration of bone quality and microarchitecture [61]. Osteoporosis in diabetes is associated with a low bone turnover, an accumulation of advanced glycation end-products, inflammation, oxidative stress, adipokine alterations, WNT dysregulation and increased bone marrow fat [65]. Thus, the evaluation of the therapeutic effect of OT in these conditions appears to be justified, as OT displays anabolic actions on bone, decreases bone marrow adiposity and may have a beneficial effect on glucose metabolism.

Although less frequent, anorexia nervosa, characterized by a marked inhibition of bone formation markers consistent with an impaired osteoblast function and low estradiol serum levels, is a secondary cause of osteoporosis [30]. In anorexia nervosa, bone marrow adiposity is increased at both axial and appendicular sites and negatively associated to BMD [66,67]. Lawson et al. showed that, in women with anorexia nervosa, nocturnal OT levels are decreased, even after controlling for estradiol levels, months of amenorrhea, the age of menarche, percent body fat or hours of exercise per week and is positively associated with a low BMD and fat mass in anorexia nervosa independently of estradiol levels [30]. Thus, bone loss induced by anorexia nervosa may be a good candidate to test the potential beneficial effects of OT, as anorexia nervosa is also characterized by altered social cognition and information processing of fear and anxiety. However, a recent meta-analysis of four publications on the effect of intranasal OT did not show any significant effect on psychiatric disorders (attentional bias and emotion recognition), but in three of the four unique studies, the patients received only a single administration of 40 IU synthetic OT, and in the fourth study, 36 IU of intra-nasal OT for 4-6 weeks. Further, there was no evaluation on bone effects [68].

Currently, there are several ways to administrate OT in human: non-invasive routes, which include intra-nasal administration, with growing evidence for increases in both peripheral and central concentrations of OT, and topical gel, that increases OT plasma levels via vaginal absorption, and invasive routes (sub-cutaneous) [56,57,58]. Thus, the intra-nasal administration of OT may be the most suitable to have all the benefits of OT, as this route increases both central and peripheral OT. However, an evaluation for safety and long-term consequences of chronic OT treatment is needed; there is only one publication on OT safety and tolerability in humans (suffering from frontotemporal dementia), which suggested that chronic intranasal OT for one week was generally well-tolerated [58,69]. The issue and the difficulty in developing OT as a treatment are to find a single route, dosage and frequency that may reach all the targets of interest in post-menopausal osteoporosis or in secondary causes of osteoporosis. There are several studies ongoing recorded in ClinicalTrials.gov phase one and phase two, testing the effects of OT treatment in mood or cognitive disorders, obesity, diabetes, including some using an oral form of OT (NCT04493515, NCT04320706), but none as a treatment for osteoporosis. The stakes are immense and research in this field is active, as shown by a recent publication by Mohan S et al., who developed enzymatically stable OT analogues with glucose lowering effects in high fat fed mice that will help to establish long term and efficient treatments as OT has a very short half-life [70]. As an outlook, to overcome these issues, OT requires proper transport systems to be delivered to the desired cells and tissues, thereby enabling the activation of the OT receptor in the target cells. In this regard, thanks to nanomedicine, the development of delivery systems represent a very active research area, including the administration of nanoparticles carrying different compounds, among which OT might be a potential candidate [71,72,73].

## 6. Conclusions

It is now well established that OT exerts an anabolic action on bone metabolism, particularly in females, as its action is amplified by estrogen, and that OT can prevent or reverse ovariectomy-induced osteoporosis in animals. However, currently there is no human data on the beneficial effects of OT as a treatment of post-menopausal osteoporosis and more generally on all menopause associated diseases. The pleiotropic actions of OT on body composition, cognitive functions, anxiety, diabetes, cardiovascular diseases and breast cancer, all concerns associated with ageing and menopause, should encourage pharmaceutical companies to invest in this promising area that represents the concept of “well ageing”.

## Figures and Tables

**Table 1:** Oxytocin and bone: human studies on oxytocin.

References	Method	Population	Biological Parameters	Bone Measure	Fragility Fracture(n)	Main Result	Key Message
Elabd S. et al. 2008 [11]	Cross sectional study	36 post-menopausal women: 20 with severe OP 16 healthy control	OT	BMD	NA	OT serum level 55% lower in OP (*p* = 0.005)	OT level is inversely correlate with the occurrence of OP.
Lawson E.A. et al. 2011 [30]	Cross sectional study	36 women: 17 with anorexia nervosa 19 healthy control	Overnight OT secretion, leptin	BMD	NA	OT serum 50% lower in anorexia nervosa (*p* = 0.003) associated with low BMD at the spine (*p* < 0.05)	Decreased nocturnal OT levels is associated with low BMD and fat mass in anorexia nervosa.
Breuil V. et al. 2011 [31]	Cross sectional study	36 post-menopausal women: 20 OP 16 healthy control Same population than Elabd S. et al. 2008 [11]	OT, estradiol, testosterone, FSH, LH, SHGB, TSH, leptin, BTM	BMD	52	OT serum level: Correlated to BMD (*p* = 0.005) Not correlated to leptin, estradiol and age	Low OT serum level is correlated to severe OP, independently of other factors associated with OP or known to regulate OT.
Lawson E.A. et al. 2013 [32]	Cross sectional study	45 women: 30 athletes: - 15 amenorrheic - 15 eumenorrheic 15 nonathletes	Overnight OT secretion	HR-pQCT (tibia, radius)	NA	Lower OT nocturnal secretion in athletes (*p* < 0.04) In amenorrheic athletes: - impaired bone microarchitecture - bone microarchitecture strongly correlated with OT (*p* < 0.05)	Important role of oxytocin for variability in bone microarchitectural and strength parameters in amenorrheic athletes.
Breuil V. et al. 2014 [33]	Cross sectional study	OPUS cohort Population-based cohort 1097 post-menopausal women 89.6% with undetectable estradiol serum level	OT, leptin, estradiol, BTM	BMD	313	High OT level associated with high BMD (*p* < 0.001) especially at the hip in women with low estradiol (*p* = 0.05) or high leptin serum level (*p* = 0.02)	OT has a direct effect on bonec ell, independently of estradiol OT mediated action.
Breuil V. et al. 2015 [34]	Cross sectional study	MINOS cohort: 552 men Age 50–85 y 265 osteopenia 32 osteoporosis	OT, BTM	BMD	60	No correlation between OT and BMD, bone turnover or prevalent fracture Weak negative relationship between OT level and fracture risk (*p* = 0.04)	Unlike women, OT level is not associated to BMD or BTM level in > 50 years old men.
Maestrini S. et al. 2018 [35]	Cross sectional study	109 women: 56 obeses (28 premenopausal) 53 normal weight (27 premenopausal)	OT	NA	NA	Lower OT serum level in post-menopausal (*p* < 0.001) obeses (*p* < 0.005) post-menopausal obeses (*p* < 0.05)	Obesity and menopause are independent negative predictors of plasma oxytocin.
Aulinas A. et al. 2021 [36]	Cross sectional study	37 Hypopituitary men: 17 with diabete insipidus 20 without diabete insipidus No osteopenia or osteoporosis	OT, vasopressin	BMD	NA	Positive association between OT serum level and BMD atm ultiplesites (*p* < 0.025) OT serum level and hip structural analysis at intertrochanteric region (*p* < 0.02)	Men with hypopituitarism and lower OT level showed lower BMD and less favorable hip geometry.

Notes: NA: Not available; OP: Osteoporosis; OT: Oxytocin; BMD: Bone mineral density; BTM: Bone turnover markers.
